# Evaluation of the Role of FGF23 in Mineral Metabolism

**DOI:** 10.4137/grsb.s2990

**Published:** 2009-08-03

**Authors:** Hiroki Yokota, João F. Raposo, Andy Chen, Chang Jiang, Hugo G. Ferreira

**Affiliations:** 1 Department of Biomedical Engineering, Indiana University Purdue University Indianapolis, Indianapolis, IN 46202, USA; 2 Endocrinology Department of the Portuguese Cancer Institute, 1099-023 Lisboa, Portugal; 3 Department of Bioengineering, University of California San Diego, La Jolla, CA 92092, USA; 4 REQUIMTE, Department of Chemistry, New University of Lisbon, 2829-516 Caparica, Portugal. Email:hyokota@iupui.edu

**Keywords:** phosphate, calcium, FGF23, calcitriol, PTH

## Abstract

Fibroblast growth factor 23 (FGF23) has recently been identified as a critical regulatory factor in phosphate (P) metabolism. Although the exact molecular mechanism of FGF23 synthesis through sensing the concentration of P is yet to be determined, experimental and clinical data indicate the influential role of FGF23 in P and calcium (Ca) homeostasis. Here, we extended our previous mathematical model in calcium regulation and examined the conceivable roles of FGF23 in mineral metabolism. We assumed that the level of FGF23 was controlled through the concentrations of P and calcitriol in serum, and its actions such as lowering of the renal threshold for P, inhibition of the production of calcitriol in the kidney tubule, and inhibition of the production of parathyroid hormone (PTH) were included. Comparisons between the models with and without FGF23 demonstrate a complex interplay of FGF23 with calcitriol and PTH. In consistent with the model, our *in vitro* experimentation indicates that expression of FGF23 is activated in the presence of P though a G-protein linked receptor. We expect that further efforts on modeling and experimental evaluation would contribute to diagnosing patients with metabolic diseases such as osteoporosis and chronic kidney diseases, and developing FGF23-linked treatment strategies.

## Introduction

The FGF family consists of 22 members for various functions in embryonic development, cellular proliferation and differentiation, tissue repair, and tumor growth and invasion. FGF23 has recently been demonstrated to represent a critical circulating hormone involved in phosphate metabolism. It is an approximately 32-kD (251 amino acids) protein and its N-terminal region contains the FGF homology domain.^1^ The primary function of FGF23 is considered to be inhibition of reabsorption of renal tubular phosphate.^2^ However, many questions are unanswered for the role of FGF23 in mineral metabolism with regards to known regulators such as PTH and calcitriol.^3^,^4^ Thus, a traditional regulatory mechanism with PTH and calcitriol needs to be rebuilt in accordance with the actions of FGF23.

Understanding the mechanism of Ca and P metabolism requires multiscale mathematical modeling. Behaviors of this highly interactive, nonlinear process can hardly be predicted by simple intuition. FGF23 is primarily synthesized in bone but its site of action is the kidney. Although genetic and biochemical analyses are essential to identify regulatory mechanisms, those approaches alone will not be sufficient to quantitatively evaluate the key processes and develop effective therapeutic interventions for patients undergoing hemodialysis and patients with metabolic disorders. Various mathematical models have been developed for calcium homeostasis.^5^–^8^ However, few models have been formulated including phosphate metabolism. Several models are proposed for bone remodeling,^9^–^11^ but mineral metabolism in the kidney is not included. To our knowledge, no comprehensive models for calcium and phosphate metabolism are available including the role of PTH and FGF23.

In this study, we included the actions of FGF23 to a previously published mathematical model of calcium and phosphate metabolism^8^ and investigated the potential influence of FGF23 on the observable state variables such as the serum concentrations of PTH, calcitriol, Ca, P, and the urinary excretion of Ca and P. In order to support the mechanism of sensing P in bone through a G-linked protein receptor, we conducted two *in vitro* experiments to evaluate the mRNA expression level of FGF23 using MLO-A5 osteocyte-like cells.^12^ The first experiment was aimed to examine whether expression of FGF23 would be elevated in the presence of P in a culture medium, while the second experiment using an inhibitor to G-linked protein receptors was designed to evaluate their potential role in sensing the level of P in bone. The specific questions addressed in the present modeling study included: How does an increase in the concentration of FGF23 affect the actions of calcitriol and PTH? And how does alteration in the concentration of calcitriol or PTH modulate the FGF23 concentration and the absorption and excretion levels of P? The model was built with and without the predicted effects of FGF23, and the dynamic responses were numerically evaluated in a transient time frame (0–100 h) as well as at 2000 h.

## Model, Materials and Methods

### Modeling dynamical exchange processes

**Figure 1:**
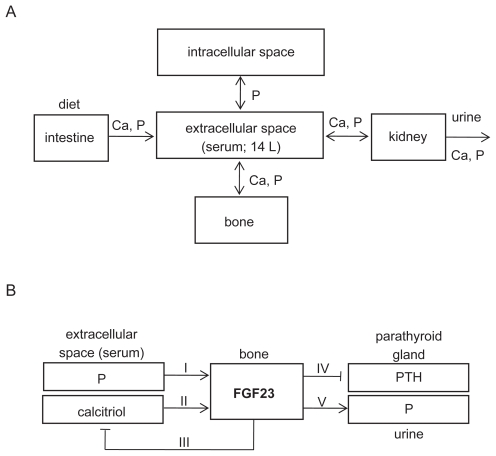
Schematic illustration of mineral metabolism in the described model with the action of FGF23. **A**) Five compartments in the model, where extracellular space contains plasma and extracellular fluid. Note that Ca = calcium, and P = phosphate. **B**) Modeling of FGF23 actions including 5 pathways (I–V).

The present work with the action of FGF23 is an extension of a previously published model without FGF23.^8^ In brief, the mineral metabolism in the previous model was treated as exchange processes at three interfaces: intracellular and extracellular spaces for P; extracellular space and bone for Ca and P; and extracellular space and the outside world (through ingestion and excretion) for Ca and P (Fig. 1A). These exchanges were controlled by two endocrine factors—PTH and calcitriol, which were assumed to be confined to the extracellular compartment.

Considered in the previous model were the functional compartments, which represented the secretory mass of the parathyroid glands, the pools of transporting intestinal translocators for Ca and P, the pools of renal tubular translocators for Ca and P, and the pool of the tubular 1-hydroxilating enzyme (converting calcidiol into calcitriol). With the exception of the parathyroid gland secretory mass that was assumed to remain constant, the dynamics of the pools were modeled with the first-order nonlinear, diffusional, differential equations. A modeling principle was that the instantaneous rate of variation of the pool size was equal to the sum of the influxes minus the sum of the effluxes. Thus, the dynamical exchange processes in each of the compartments was represented by a time derivative of 8 pools in a general form:

(1) $$d(Qx)/dt=\sum J_{\text{influx}}(x)-\sum J_{\text{outflux}}(x)$$

where *Q* = size of the pool of the state variable *x* (e.g. Ca in bone); *J*(*x*)_influx_ = influxes of variable x (e.g. intestinal uptake of P); and *J*(*x*)_outflux_ = outfluxes (e.g. urinary output of Ca). These 8 pools included 3 pools of Ca in bone, extracellular space (serum), and intestine, 4 pools of P in bone, serum, intracellular space, and kidney, and 1 pool of PTH in parathyroid grand. No independent state variables were considered for Ca in kidney or intracellular space, or for P in intestine (see details in 8 and Appendix).

### Modeling five FGF23-linked pathways

In addition to dynamical alterations of three molecular regulators (PTH, calcitriol, and 1α-hydroxylase) in the previous model, the dynamical alteration of FGF23 was added in the current model as a mineral metabolism regulator (Fig. 1B). The total amount of FGF23 in serum, *Q*_FGF23_ (pg), was modeled:

(2) $$d(Q_{\text{FGF}23})/dt=J_{\text{FGF}23}-\delta \cdot Q_{\text{FGF}23}$$

where *J*_FGF23_ = rate of FGF23 secretion (pmole/hr), and *δ* = rate factor for FGF23 degradation (0.8 h^−1^). Since the secretion of FGF23 is reported to be modulated by the serum concentrations of P and calcitriol (pathways I and II in Fig. 1B, respectively), *J*_FGF23_ was modeled:

(3) $$J_{\text{FGF}23}(X_{P},X_{cal})=\{V_{P}\cdot X_{P}/(X_{P}+K_{P})\}\{V_{cal}\cdot X_{cal}/(X_{cal}+K_{cal})\}$$

in which *X_P_*, and *X_cal_* were the serum concentrations of *P*, and calcitriol. The parameters (*V_P_*, *V_cal_*) and (*K_P_*, *K_cal_*) represented the maximum rates and Michaelis constants in Michaelis-Menten kinetics, respectively.

Three effects of FGF23 were considered in the extended model: inhibition of the production of calcitriol in the kidney tubule (pathway III), inhibition of the production of PTH by the parathyroid glands (pathway IV), and lowering of the renal threshold for *P* (pathway V). In each pathway, the effect of FGF23 was modeled in a form:

(4) $$\begin{array}{l} V_{\text{max}}\cdot X_{\text{FGF}23}/(X_{\text{FGF}23}+K_{\text{M}.\text{M}.}),\\ V_{\text{max}}\cdot K_{\text{M}.\text{M}}/(X_{\text{FGF}23}+K_{\text{M}.\text{M}.}) \end{array}$$

in which *V*_max_ = maximum rate in Michaelis-Menten kinetics; *K*_M.M._ = affinity for the FGF23 receptor; and *X*_FGF23_ = serum concentration of FGF23. The former was used for stimulatory effects (pathway V) and the latter for inhibitory (pathways III and IV).

### Dynamical simulation

Prior to numerical simulations, we identified the reference states (steady-states), in which the state variables were kept constant. Using stepwise perturbations to the selected reference state variables, we evaluated the transient responses for 0–100 h, and a set of new steady states at 2000 h after an onset of perturbations. In order to avoid an impulsive jerk, stepwise perturbation was given using an exponential function in the initial perturbation phase with a time constant of 1 h. The key parameters employed in the study are listed (Table 1).

### Analysis of FGF23 expression

Using MLO-A5 osteocyte-like cells, we conducted two experiments for investigation of FGF23 mRNA expression. In the first experiment, cells were grown either in the presence or absence of hydroxylapatite [Ca_10_(PO_4_)_6_(OH)_2_] and examined the effects of hydroxylapatite in the culture medium on the FGF23 mRNA level. In the second experiment, cells were grown with and without a pharmacological agent (pertussis toxin; an inhibitor of G-protein linked receptors)^13^ and tested whether expression of FGF23 would be affected by blocking a G-protein linked receptor as a potential phosphate sensor in bone cells.

In both experiments, approximately 1 × 10^6^ cells were seeded on the 3D collagen matrix (20 mm × 40 mm × 2 mm; Zimmer Dental) and cultured in αMEM medium containing 5% fetal bovine serum, 5% bovine serum and antibiotics (50 units/ml penicillin and 50 μg/ml streptomycin; Invitrogen) for two days. Deposition of hypdroxylapatite was conducted by immersion of the collagen matrix in 500 mM Na_2_HPO_4_ solution for 5 min followed by rinsing in water and immersion in 500 mM CaCl_2_ solution for 5 min.^14^ The matrix was thoroughly rinsed in water. Pertussis toxin (Calbiochem) was administered for 1 day at a concentration of 100 or 500 ng/ml.

Total RNA was harvested using RNeasy mini kits (Qiagen), and reverse transcription was conducted with high capacity cDNA reverse transcription kits (Applied Biosystems).^15^ Quantitative real-time PCR was performed using ABI 7500 with Power SYBR green PCR master mix kits (Applied Biosystems). The PCR primers were FGF23 (5′-GACCAGCTATCACCTACAGATCCAT-3′; 5′-TGTAATCATCAGGGCACTGTAGATG-5′), and GAPDH (5′-TGCACCACCAACTGCTTAG-3′; 5′-GGATGCAGGGATGATGTTC-3′).

The mRNA level of GAPDH was used as an internal control. In evaluation of the effect of hydroxylapatite on FGF23 expression, the GAPDH mRNA levels in the two samples were compared and the ratio (GAPDH level in hydroxylapatite/GAPDH level in control) was calculated. The level of FGF23 mRNA in hydroxylapatite was then divided by this ratio, and the mean value of the FGF23 mRNA level in control was set to 1. In evaluation of the effect of pertussis toxin on FGF23 expression, the same normalization procedure based on the GAPDH mRNA levels was taken in which the FGF23 mRNA level for the sample with 0 ng/ml pertussis toxin was set to 1. Experiments were conducted three times.

## Results

### Responses to a stepwise increase in FGF23 secretion

**Figure 2:**
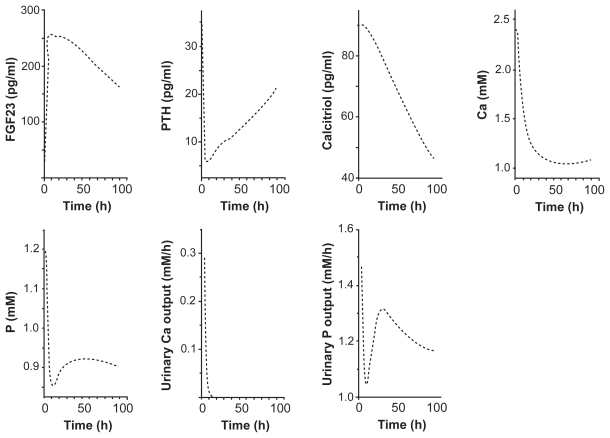
Transient responses (0–100 h) to a stepwise increase in FGF23. The variables include the concentrations of FGF23, PTH, calcitriol, calcium, and phosphate in serum, and the excretion levels of calcium and phosphate in urine.

Based on the above mentioned formulation of the expression and actions of FGF23, we first evaluated the effects of the stepwise increase in the secretion of FGF23 on the regulatory and homeostatic variables such as the concentrations of PTH, calcitriol, Ca, and P in serum as well as the absorption and excretion amounts of Ca and P. In the transient responses (0–100 h) to an increase in FGF23 from 30 to 260 pg/ml, those variables were decreased with varying temporal behaviors (Fig. 2). The concentration of PTH, for instance, was swiftly reduced within a few h followed by a gradual recovery, while the concentration of calcitriol was monotonously lowered. The concentrations of Ca and P in serum were steeply dampened in the initial ~10 h and they stayed at the lower levels. Interestingly, the maximum reduction was observed in the excretion amount of Ca, which became nearly zero in 10 h.

**Figure 3:**
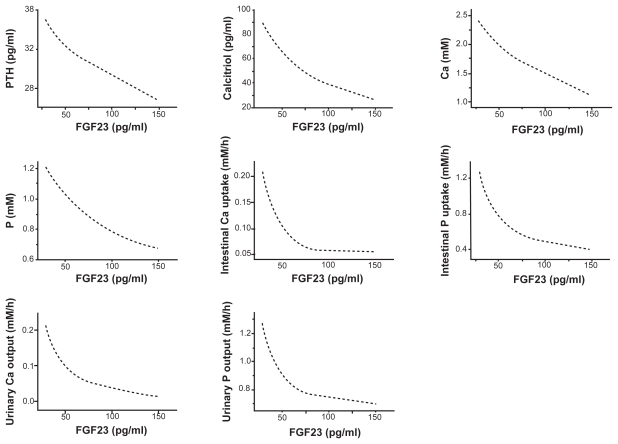
Steady-state responses (2000 h) to a stepwise increase in FGF23. The variables include the concentrations of PTH, calcitriol, calcium, and phosphate in serum, the absorption levels of calcium and phosphate in intestine, and the excretion levels of calcium and phosphate in urine.

In order to characterize the chronic effects of the increase in FGF23, we evaluated the pseudo steady-state values (2000 h) (Fig. 3). The variables as a function of the level of FGF23 including the concentrations of PTH, calcitriol, Ca, and P in serum, and the absorption and excretion levels of Ca and P exhibited a monotonous decrease as the level of FGF23 increased.

### Responses to a stepwise increase in the uptake of P

**Figure 4:**
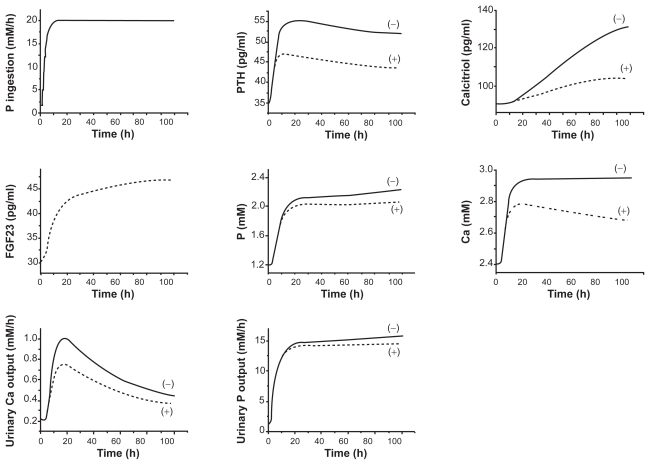
Transient responses (0–100 h) to a stepwise increase in P. The dotted and solid curves correspond to the responses with and without the action of FGF23. The variables include the stepwise increase in P, the concentrations of PTH, calcitriol, FGF23, phosphate, and calcium in serum, and the excretion levels of calcium and phosphate in urine.

To evaluate the effects of FGF23 actions in mineral metabolism, we next evaluated the responses of the state variables in the presence and absence of FGF23 actions after a stepwise increase in the phosphate intake. As predicted in the responses to the stepwise FGF23 increase in Figures 2 and 3, the transient responses revealed that the model with FGF23 always reduced the levels of 7 state variables within 20 h (Fig. 4). The concentrations of PTH, calcitriol, Ca, and P in serum, for instance, were lowered by 17%, 23%, 9%, and 10% in 100 h, respectively.

**Figure 5:**
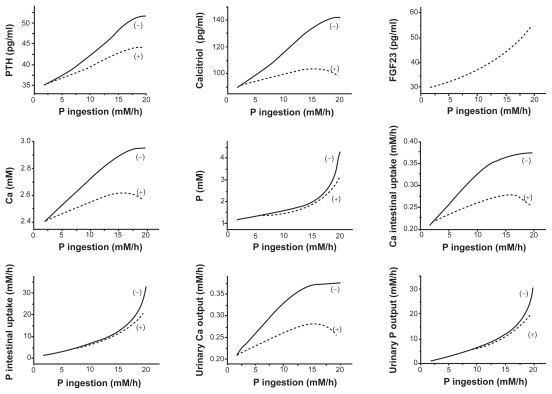
Steady-state responses (2000 h) to a stepwise increase in P. The dotted and solid curves correspond to the responses with and without the action of FGF23. The variables include the concentrations of PTH, calcitriol, FGF23, calcium, and phosphate in serum, the absorption levels of calcium and phosphate in intestine, and the excretion levels of calcium and phosphate in urine.

Consistent with the transient responses, those variables (the concentrations of PTH, calcitriol, Ca, P in serum, the absorption levels of calcium and phosphate in intestine, and the excretion levels of calcium and phosphate in urine) at 2000 h after the onset of the increased phosphate intake in diet from 2 to 20 mM/h were significantly lowered in the presence of FGF23 (Fig. 5). The observed reductions were more evident in the responses to a higher intake level of P.

### Responses to a stepwise increase in Ca uptake

**Figure 6:**
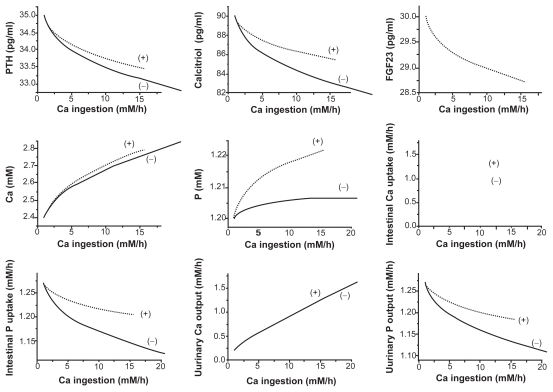
Steady-state responses (2000 h) to a stepwise increase in the absorption level of calcium. The dotted and solid curves correspond to the responses with and without the action of FGF23. The variables include the concentrations of PTH, calcitriol, FGF23, calcium, and phosphate in serum, the absorption levels of calcium and phosphate in intestine, and the excretion levels of calcium and phosphate in urine.

The effects of FGF23 in response to the increased uptake of Ca were opposite to those in response to the elevated uptake of P, since the stepwise increase in Ca uptake lowered the concentration of FGF23 in serum (Fig. 6). Although the FGF23 model presented virtually no influence on the absorption or excretion of Ca, it elevated the intestinal uptake and the urinary output of P. Furthermore, in the presence of FGF23 actions the concentrations of PTH, calcitriol, Ca, and P in serum were elevated.

### Responses to a stepwise increase in calcitriol or PTH

**Figure 7:**
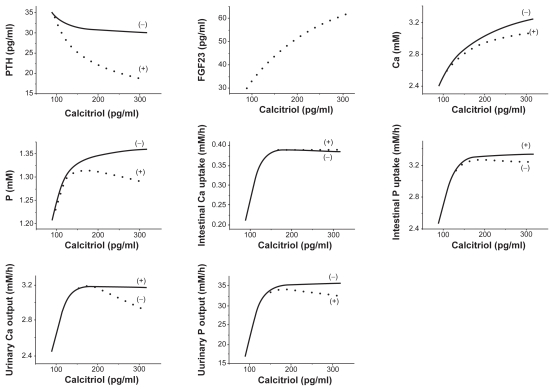
Steady-state responses (2000 h) to a stepwise increase in the level of calcitriol in serum. The dotted and solid curves correspond to the responses with and without the action of FGF23. The variables include the concentrations of PTH, FGF23, calcium, and phosphate in serum, the absorption levels of calcium and phosphate in intestine, and the excretion levels of calcium and phosphate in urine.

**Figure 8:**
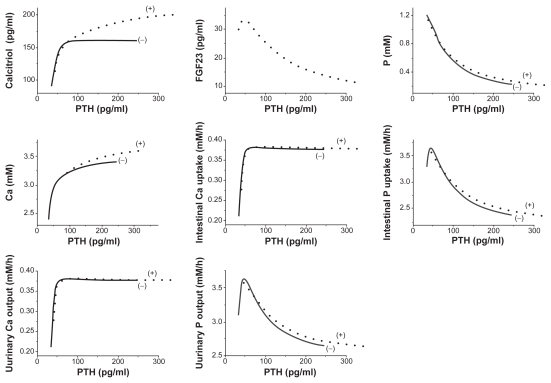
Steady-state responses (2000 h) to a stepwise increase in the concentration of PTH in serum. The dotted and solid curves correspond to the responses with and without the action of FGF23. The variables include the concentrations of calcitriol, FGF23, phosphate, and calcium in serum, the absorption levels of calcium and phosphate in intestine, and the excretion levels of calcium and phosphate in urine.

Numerical simulations were also conducted in response to a stepwise increase in two regulatory factors (calcitriol or PTH), and the pseudo steady-state values (2000 h) in the presence and absence of FGF23 were evaluated. Although the synthesis of FGF23 is affected by calcitriol and not by PTH in the current model, the stepwise perturbations in both calcitriol and PTH altered the level of FGF23. First, the increase in calcitriol upregulated the level of FGF23 (Fig. 7), while that in PTH downregulated in (Fig. 8). Second, compared to the model without the actions of FGF23 the stepwise elevation of calcitriol in the FGF23 model reduced the level of PTH. On contrary, the stepwise increase of PTH in the current model significantly elevated the concentration of calcitriol in serum.

Neither perturbation did not show any clear effects on the absorption of Ca in intestine, but they changed the absorption and excretion of P. In accordance with the predicted increase in FGF23, the stepwise input of calcitriol decreased the intestinal uptake and the urinary output of P. In concert to the simulated decrease in FGF23, on the other hand, the stepwise elevation of PTH increased the intestinal absorption and the urinary excretion of P.

### Expression of FGF23 in hydroxylapatite-deposited collagen matrix

**Figure 9:**
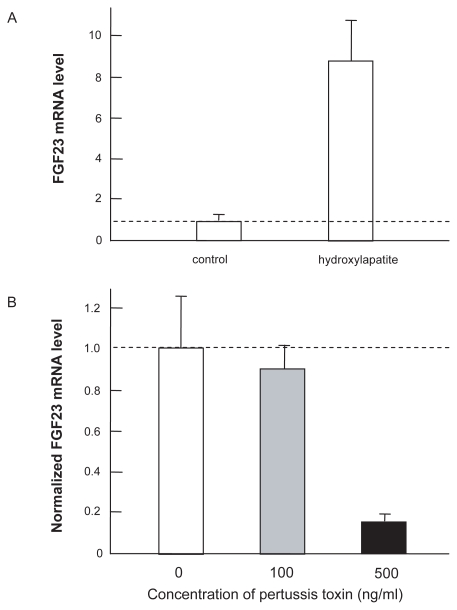
Expression of FGF23 mRNA in MLO-A5 cells. **A**) Elevated expression in response to hydroxylapatite in 3D collagen matrix. The dashed line shows the control level without hydroxylapatite. **B**) Reduced FGF23 expression by pertussis toxin (100 ng/ml, and 500 ng/ml) in 3D collagen matrix in the presence of hydroxylapatite.

The FGF23 mRNA level was unregulated in the culture that was rich in Ca and phosphate (hydroxylapatite). Quantitative real-time PCR showed that the mRNA level of FGF23 was elevated 8.7 fold in the matrix deposited with hydroxylapatite (Fig. 9A). Furthermore, the elevated FGF23 mRNA level in hydroxylapatite was suppressed by the inhibitor of G-protein linked receptors approximately by 10% and 85% at the concentrations of 100 and 500 ng/ml pertussis toxin, respectively (Fig. 9B).

## Discussion

The current study presented mathematical formulation of the metabolism of Ca and P including the actions of FGF23. Prior to the discovery of FGF23, the calcium-PTH-calcitriol axis has been considered a primary regulatory pathway in mineral metabolism. The major function of this axis is maintenance of the serum calcium level, while its role in the serum phosphate level is treated secondarily.^16^ Recent advances in our understanding of disorders involving mineral metabolism have led to the FGF23-bone-kidney axis being added to the calcium-PTH-calcitriol pathway.^17^ In response to an increase in the phosphate level in serum (pathway I), it is proposed that the expression of FGF23 is elevated in osteocytes in bone. However, no phosphate sensor has been identified. The present experimentation for the first time activated expression of FGF23 in osteocyte cells *in vitro*, although hydroxylapatite is a compound consisting of calcium and phosphate. Furthermore, similarly to a calcium sensor,^18^ the mRNA expression analysis using pertussis toxin indicates a possibility that a phosphate sensor is also a G-protein linked receptor.

The described model allows prediction of the concentrations of FGF23, calcitriol, PTH, Ca, and P in serum together with the intestinal absorption and the urinary excretion of Ca and P in response to varying perturbations. The FGF23 elevation is considered to reduce production of calcitriol in the kidney and decrease absorption of calcium and phosphate from the intestine.^19^ Although the exact molecular mechanism is yet to be determined, numerical simulations revealed that the presence of FGF23 altered the levels of both Ca and P in serum through a complex interplay with calcitriol and PTH (pathways II–IV). Calcitriol is modeled to be stimulatory to the circulatory level of FGF23 in serum (pathway II), while PTH is predicted to be inhibitory in an indirect fashion.

The current model should be further refined using experimental and clinical data regarding the upstream and downstream events for expression of FGF23. Furthermore, the parameter values in Table 1 need validations. Nevertheless, the recent clinical studies support that FGF23 inhibits renal production of calcitriol (pathway III), downregulates PTH release (pathway IV), and induces renal phosphaturia (pathway V).^20^ Furthermore, FGF23 and PTH in serum are shown to be associated *in vivo*, supporting the assumption of the model that FGF23 directly regulates PTH expression.^21^ Using both rats and *ex vivo* rat parathyroid cultures, it is reported that FGF23 suppresses expression and secretion of PTH.^22^

Physiological data from FGF23 mutations can be also used for validation of the role of FGF23. The FGF23 gene was identified by its mutations associated with autosomal dominant hypophosphatemic rickets, which is an inherited phosphate wasting disorder.^17^ Thereafter, a variety of disorders resulting from FGF23 malfunctioning have been reported. These disorders, which are caused by mutations in the genes that directly or indirectly interact with FGF23, include hyperphosphatemic familial tumoral calcinosis, hereditary hypophosphatemic rickets with hypercalciuria, autosomal recessive hypophosphatemic rickets, and X-linked dominant hypophosphatemic rickets. Furthermore, clinical data from patients with chronic kidney diseases should be useful in evaluating the interactions among calcitriol, PTH, and FGF23.

In summary, the described mathematical model allows us evaluation of the dynamical metabolic processes in Ca and P by considering the regulatory actions of calcitriol, PTH, and FGF23. Biological experimentation indicated a potential role of G-linked protein receptors in regulating expression of FGF23 in bone cells. Further evaluations are necessary to refine the model. The results herein support that the described model-based approach together with biological verification is useful for characterization of the dynamical metabolic responses, which is indispensable for development of quantitative treatment strategies for patients with metabolic disorders.

## Figures and Tables

**Table 1:** FGF23-linked parameters employed in the study.

FGF23 pathways	Effect	Remark	Value
**General**		Rate of FGF23 degradation	0.8 h^−1^
		
		Reference FGF23 concentration	0.03 ng/ml

I (P to FGF23)	stimulatory	Michaelis constant	0.03 mg/ml
II (calcitriol to FGF23)	stimulatory	Michaelis constant	0.038 ng/ml
III (FGF23 to calcitriol)	inhibitory	Michaelis constant	0.03 ng/ml
IV (FGF23 to PTH)	inhibitory	Michaelis constant	0.03 ng/ml
V (FGF23 to P)	stimulatory	Michaelis constant	0.03 ng/ml

## Appendix

According to our previous model without considering the effects of FGF23,^8^ the rate of changes of the calcium pools in bone (*Ċ**_b_*) and plasma including extracellular fluid (*Ċ**_p_*) as well as the transportable fraction in intestine(*Ċ**_i_**^f^*) are expressed:

(A1) $${\dot{C}}_{b}(t)=Q_{pb}^{c}-Q_{bp}^{c}$$

(A2) $${\dot{C}}_{p}(t)=(Q_{bp}^{c}+Q_{ip}^{c})-(Q_{pb}^{c}+Q_{pu}^{c})$$

(A3) $${\dot{C}}_{i}^{f}(t)=K_{1}(1-C_{i}^{f})-K_{2}C^{f}$$

Where *Q**_xy_**^c^* = rate of a calcium transfer from tissue “x” to tissue “y”. The dynamical changes of the phosphorus pools in bone (*Ṗ**_b_*), intracellular fluid (*Ṗ**_c_*), and plasma (*Ṗ**_p_*) together with the transportable fraction in the kidney (*Ṗ**_k_**^f^*) are expressed:

(A4) $${\dot{P}}_{b}(t)=Q^{P}pb-Q^{P}bp$$

(A5) $${\dot{P}}_{c}(t)=Q_{pc}^{p}-Q_{cp}^{p}$$

(A6) $${\dot{P}}_{k}^{f}(t)=G\{K_{5}(1-P_{k}^{f})-K_{6}P_{k}^{f}\}$$

(A7) $${\dot{P}}_{p}(t)=(Q_{bp}^{p}+Q_{cp}^{p}+Q_{ip}^{p})-(Q_{pb}^{p}+Q_{pc}^{p}+Q_{pu}^{p})$$

in which *Q**_xy_**^p^* = rate of a phosphorus transfer from tissue “*x*” to tissue “*y*”. Lastly, the changes of 1α-hydroxylase (*ḣ**_k_*), calcitriol (*v̇**_p_*), and PTH together with a dynamic alteration of secretory mass of the parathyroid gland (*π̇**_p_* and *π̇**^f^*) are modeled:

(A8) $${\dot{h}}_{k}(t)=s_{h}-\delta_{h}h_{k}$$

(A9) $${\dot{v}}_{p}(t)=s_{v}h_{k}-\delta_{v}v_{p}$$

(A10) $${\dot{\pi }}_{p}(t)=s_{\pi }-\delta_{\pi }\pi_{p}$$

(A11) $${\dot{\Pi }}^{f}(t)=K_{3}(1-\Pi^{f})-K_{4}\Pi^{t}$$

The reference values in the above formulation are listed below assuming that the volume of plasma and extracellular fluid is 14 L.

**Table:**

*C**_b_*	Exchangeable pool amount of **C**alcium in “bone (**b**)”	4.00 g
*C*_p_	Pool amount of **c**alcium in“plasma and extracellular fluid (**p**)”	1.34 g
*C**_i_**^f^*	transportable **C**alcium **f**raction in “intestine (**i**)”	0.500
*P**_b_*	Exchangeable pool amount of **P**hosphorus in “bone (**b**)”	1.86 g
*P**_c_*	Pool amount of **P**hosphorus in “cells (**c**)”	104 g
*P**_p_*	Pool amount of **P**hosphorus in “plasma and extracellular fluid (**p**)”	0.518 g
*P**_k_**^f^*	Transportable **P**hosphorus **f**raction in “kidney (**k**)”	0.500
*V**_p_*	Concentration of calcitriol [1,25(OH)_2_**v**itamin D] in “plasma and extracellular fluid(**p**)”	37.5pg/ml
*π**_p_*	concentration of **p**th in “plasma and extracellular fluid(**p**)”	36.4pg/ml
